# Assessment of Annual Cost of Substance Use Disorder in US Hospitals

**DOI:** 10.1001/jamanetworkopen.2021.0242

**Published:** 2021-03-05

**Authors:** Cora Peterson, Mengyao Li, Likang Xu, Christina A. Mikosz, Feijun Luo

**Affiliations:** 1 National Center for Injury Prevention and Control, Centers for Disease Control and Prevention, Atlanta, Georgia

## Abstract

**Importance:**

A persistently high US drug overdose death toll and increasing health care use associated with substance use disorder (SUD) create urgency for comprehensive estimates of attributable direct costs, which can assist in identifying cost-effective ways to prevent SUD and help people to receive effective treatment.

**Objective:**

To estimate the annual attributable medical cost of SUD in US hospitals from the health care payer perspective.

**Design, Setting, and Participants:**

This economic evaluation of observational data used multivariable regression analysis and mathematical modeling of hospital encounter costs, controlling for patient demographic, clinical, and insurance characteristics, and compared encounters with and without secondary SUD diagnosis to statistically identify the total attributable cost of SUD. Nationally representative hospital emergency department (ED) and inpatient encounters from the 2017 Healthcare Cost and Utilization Project Nationwide Emergency Department Sample and National Inpatient Sample were studied. Statistical analysis was performed from March to June 2020.

**Exposures:**

*International Statistical Classification of Diseases, Tenth Revision, Clinical Modification (ICD-10-CM)* principal or secondary SUD diagnosis on the hospital discharge record according to the Clinical Classifications Software categories (disorders related to alcohol, cannabis, hallucinogens, inhalants, opioids, sedatives, stimulants, and other substances).

**Main Outcomes and Measures:**

Annual attributable SUD medical cost in hospitals overall and by substance type (eg, alcohol). The number of encounters (ED and inpatient) with SUD diagnosis (principal or secondary) and the mean cost attributable to SUD per encounter by substance type are also reported.

**Results:**

This study examined a total of 124 573 175 hospital ED encounters and 33 648 910 hospital inpatient encounters from the 2017 Healthcare Cost and Utilization Project Nationwide Emergency Department Sample and National Inpatient Sample. Total annual estimated attributable SUD medical cost in hospitals was $13.2 billion. By substance type, the cost ranged from $4 million for inhalant-related disorders to $7.6 billion for alcohol-related disorders.

**Conclusions and Relevance:**

This study’s results suggest that the cost of effective prevention and treatment may be substantially offset by a reduction in the high direct medical cost of SUD hospital care. The findings of this study may inform the treatment of patients with SUD during hospitalization, which presents a critical opportunity to engage patients who are at high risk for overdose. Aligning incentives such that prevention cost savings accrue to payers and practitioners that are otherwise responsible for SUD-related medical costs in hospitals and other health care settings may encourage prevention investment.

## Key Points

**Question:**

How much does substance use disorder cost each year in US hospitals?

**Findings:**

In this economic evaluation of 124 573 175 hospital emergency department encounters and 33 648 910 hospital inpatient encounters, the annual medical cost associated with substance use disorder in US emergency departments and inpatient settings exceeded $13 billion in 2017.

**Meaning:**

These findings suggest that costs associated with substance use disorder are high; costs of treatment and prevention could potentially be offset by reducing the direct medical cost of substance use disorder.

## Teaser

This economic evaluation investigates the annual estimated medical cost of substance use disorder in US emergency departments and inpatient settings.

## Introduction

The US drug overdose death rate has more than tripled in 2 decades, reaching more than 70 000 deaths in 2019.^1^ In the most recent available data, hospital admissions with principal diagnosis of mental health or substance use disorder (SUD) increased 12% from 2005 to 2014 and emergency department (ED) visits increased 44% from 2006 to 2014.^2,3^ Hospital encounters with SUD as a concomitant condition (not principal diagnosis) are also increasing; admissions documenting patients’ opioid use disorder without overdose quadrupled from 1993 to 2016 (to 155 discharges per 100 000 population).^4^

These trends create urgency to estimate attributable direct costs to assist in identifying cost-effective ways to prevent SUD and link people to effective treatment. Previous analysis has addressed the prevalence and mean cost of hospital encounters that include mental health or SUD diagnosis.^5^ Decision-making about SUD prevention investment can benefit more from the estimated total cost of hospital care that is attributable to SUD—that is, the cost that potentially could be minimized through successful prevention or treatment. The attributable cost of SUD in US hospitals can be derived through person-based statistical models of medical costs, which compare patients with and without a health condition.^6^ This study aimed to use nationally representative data to estimate the attributable direct annual medical cost of SUD in US hospitals.

## Methods

This economic evaluation did not require institutional board review or informed patient consent because all data were publicly available and no human participants were involved, per 45 CFR part 46. This study followed the relevant sections of the Consolidated Health Economic Evaluation Reporting Standards (CHEERS) reporting guideline.^7^

We analyzed the 2017 Healthcare Cost and Utilization Project Nationwide Emergency Department Sample (HCUP-NEDS) and National Inpatient Sample (HCUP-NIS), which offer survey-weighted national estimates of community hospital encounters based on discharge records. Diagnoses by *International Statistical Classification of Diseases, Tenth Revision, Clinical Modification (ICD-10-CM)* code, including SUD (Table 1), were classified by Clinical Classification Software Refined groups. ED records indicating admission to the same hospital and inpatient records indicating transfer admission from another hospital were excluded to avoid double counting. Elixhauser Comorbidity Software identified patient comorbidities.^8^ The main outcome measures were the total annual attributable SUD medical cost in hospitals overall and by substance type (eg, alcohol). The number of encounters (ED and inpatient) with SUD diagnosis (principal or secondary) and the mean cost attributable to SUD per encounter by substance type are also reported. One-year cost estimates as 2017 US dollars approximate the health care payer perspective; discounting was not relevant because of the 1-year time horizon.

**Table 1.** Substance Use Disorder Definition

Substance	CCSR Code	CCSR Description	*ICD-10-CM* code
Alcohol	MBD017	Alcohol-related disorders	F10,^a^ G312, G621, I426, K292, K70, O354, O9931
Cannabis	MBD019, MBD030,^b^ MBD034^c^	Cannabis-related disorders	F12,^a^ T407^d^
Hallucinogen	MBD022, MBD031,^b^ MBD034^c^	Hallucinogen-related disorders	F16,^a^ T408,^d^ T409^d^
Inhalant	MBD023, MBD033,^b^ MBD034^c^	Inhalant-related disorders	F18,^a^ T410^d^
Opioid	MBD018, MBD028,^b^ MBD034^c^	Opioid-related disorders	F11,^d^ T400,^d^ T401,^d^ T402,^d^ T403,^d^ T404,^d^ T406^d^
Sedative	MBD020, MBD032, MBD034^b^^,^^c^	Sedative-related disorders	F13,^a^ T426,^d^ T427^e^
Stimulant	MBD021, MBD029,^b^ MBD034^c^	Stimulant-related disorders	F14,^a^ F15,^a^ T405,^d^ T436^d^
Other	MBD025	Other specified substance-related disorders^f^	F19,^a^ O355, O9932

Abbreviations: CCSR, Clinical Classifications Software Refined version 2020.2 for *ICD-10-CM* diagnoses; *ICD-10-CM*, *International Statistical Classification of Diseases, Tenth Revision, Clinical Modification*.

^a^ This includes all codes in the series except for in remission (fifth digit equal to 1).

^b^ These were subsequent encounters.

^c^ Only sequela diagnoses from the listed substance (eg, stimulant) were included from CCSR MBD034.

^d^ Includes all codes in the series except for assault (sixth digit equal to 3) and underdosing (sixth digit equal to 6).

^e^ Includes all codes in the series except for assault (fifth digit equal to 3) and underdosing (fifth digit equal to 6).

^f^ Includes abuse or complications from other psychoactive substances and maternal care for drug use complicating pregnancy and childbirth.

HCUP-NEDS and HCUP-NIS report hospital facility charges per encounter. The estimated medical cost per encounter was calculated as the facility charge multiplied by a cost to charge ratio (CCR) and a professional fee ratio (PFR). HCUP provides CCR estimates based on hospital accounting reports from the Centers for Medicare & Medicaid Services to translate hospital facility charges to actual expenses incurred in the production of hospital services, such as wages, supplies, and utilities.^9^ Mean CCR among analyzed inpatient encounters was 0.293 (data not shown), suggesting hospitals’ facility cost was approximately 30% of the facility charge. CCR was estimated for ED records (mean: 0.385, data not shown) by matching HCUP-NEDS hospital characteristics to HCUP-NIS CCR data.^10^ PFR estimates in the reference source were based on insurance payments to physicians relative to facility payments in medical claims data.^11^ PFR was assigned by encounter type and primary payer for this analysis: ED (Medicaid or Medicare, 1.440; all other payers, 1.286) or inpatient (Medicaid or Medicare, 1.177; all other payers, 1.264).^11^ For example, this means the estimated facility cost of a Medicaid ED encounter was increased by 44% to account for professional fees. A $70 ambulance cost was also assigned to ED encounters (a mean expected value based on the fact that 14.5% of ED visits have ambulance arrival at a mean cost $479 as 2017 US dollars).^12,13,14^ The provenance of this study’s cost data supports monetary results presented in terms of medical costs, rather than payments or reimbursements (which are relevant terms when financial transactions from medical claims constitute the primary basis for estimated medical costs).

### Statistical Analysis

These results reflect appropriate reweighting after excluding records (10% of eligible) with missing data (charges, diagnosis code, sex, age, primary payer, disposition, or length of inpatient stay). The associated medical cost of SUD overall and by substance type was calculated using discretely estimated adjusted mean associated costs of principal and secondary SUD diagnoses from 2 multivariable generalized linear models of total encounter cost, controlling for patient demographic, clinical, and insurance characteristics. Statistical analysis was conducted with SAS version 9.4 (SAS Institute) and Stata version 16 (StataCorp) from March to June 2020.

Model 1 included only encounters with principal SUD diagnosis (eg, drug overdose) and controlled for all secondary SUD diagnoses. The mean of that model’s estimated values (using Stata’s margins command) was the estimated adjusted mean cost of an encounter with a principal SUD diagnosis (Table 2).

**Table 2.** Annual Cost of SUD in US Hospitals, 2017^a^

Encounter type and SUD diagnosis type	Total	Substance							
		Alcohol	Cannabis	Hallucinogen	Inhalant	Opioid	Sedative	Stimulant	Other
ED, encounters, No. (95% CI)^b^^,^^c^									
None	119 586 136 (112 785 578 to 126 386 695)	NA	NA	NA	NA	NA	NA	NA	NA
Any	4 987 039 (4 637 814 to 5 336 263)	2 517 498 (2 334 469 to 2 700 527)	902 682 (802 926 to 1 002 437)	30 446 (24 018 to 36 874)	6006 (4528 to 7484)	831 833 (756 006 to 907 660)	125 819 (115 388 to 136 249)	765 284 (684 311 to 846 257)	592 776 (532 904 to 652 648)
Principal	2 171 056 (1 997 679 to 2 344 433)	1 284 278 (1 172 718 to 1 395 838)	69 708 (64 172 to 75 243)	15 231 (11 877 to 18 585)	2959 (1 927 to 3 992)	393 045 (353 443 to 432 648)	43 372 (40 363 to 46 381)	176 365 (154 705 to 198 025)	186 097 (165 608 to 206 586)
Secondary	3 265 288 (2 996 740 to 3 533 837)	1 360 122 (1 238 480 to 1 481 763)	836 763 (738 762 to 934 763)	16 033 (12 532 to 19 535)	3093 (2226 to 3961)	492 968 (442 250 to 543 686)	84 311 (75 967 to 92 655)	606 166 (539 398 to 672 934)	414 591 (367 678 to 461 504)
Inpatient, encounters, No. (95% CI)^b^^,^^c^									
None	30 267 795 (29 748 856 to 30 786 733)	NA	NA	NA	NA	NA	NA	NA	NA
Any	3 381 115 (3 298 349 to 3 463 881)	1 631 331 (1 590 181 to 1 672 482)	771 974 (745 658 to 798 290)	16 933 (15 137 to 18 729)	4061 (3149 to 4972)	919 339 (888 577 to 950 101)	174 056 (164 715 to 183 397)	664 338 (636 185 to 692 492)	338 137 (324 723 to 351 550)
Principal	660 016 (629 490 to 690 541)	394 461 (377 761 to 411 161)	9844 (9089 to 10 599)	2217 (1936 to 2497)	136 (83 to 189)	141 007 (128 180 to 153 834)	21 814 (20 267 to 23 362)	54 898 (51 511 to 58 286)	35 638 (32 243 to 39 034)
Secondary	3 147 249 (3 070 429 to 3 224 068)	1 431 088 (1 397 293 to 1 464 883)	764 508 (738 393 to 790 623)	15 285 (13 611 to 16 960)	3945 (3038 to 4852)	809 200 (783 786 to 834 613)	153 446 (145 130 to 161 762)	631 995 (605 058 to 658 931)	306 257 (294 084 to 318 429)
SUD cost per encounter (2017), $ (95% CI)^d^									
ED									
Principal	1985 (1893 to 2077)	2082 (1982 to 2183)	1781 (1675 to 1886)	1677 (1493 to 1862)	1317 (1187 to 1446)	1736 (1642 to 1830)	1815 (1704 to 1926)	2058 (1944 to 2171)	1860 (1746 to 1975)
Secondary	740 (632 to 848)	773 (628 to 918)	491 (375 to 606)	419 (36 to 803)	NS	509 (328 to 690)	620 (316 to 923)	385 (276 to 494)	483 (330 to 637)
Inpatient									
Principal	9693 (9361 to 10 025)	9806 (9353 to 10 259)	8014 (7484 to 8545)	9204 (8709 to 9699)	NC	9068 (8678 to 9458)	8381 (8111 to 8652)	9690 (9336 to 10 045)	5704 (5476 to 5931)
Secondary	NS	NS	165 (36 to 294)	NS	NS	NS	374 (46 to 703)	504 (294 to 713)	NS
Total SUD cost, millions (2017), $^e^	13 170	7593	740	53	4	2212	371	1447	750

Abbreviations: ED, emergency department; NA, not applicable; NC, not calculated because of small sample size; NS, not significantly greater than $0 cost; SUD, substance use disorder.

^a^ This table’s data are sourced from the Healthcare Cost and Utilization Project Nationwide Emergency Department Sample and National Inpatient Sample.

^b^ ED encounters are visits with treat and release or fatality disposition; inpatient encounters are all admissions, including those originating in an ED and those ending in fatality, except transfers from another acute care hospital.

^c^ These are survey-weighted estimates. Encounters could have both principal and secondary SUD diagnosis or more than one substance type; therefore, “Any” is not a sum of “Principal” and “Secondary” measures and “Total” is not a sum of “Substance type” measures.

^d^ Results are marginal effect estimates from generalized linear models of the total cost of hospital encounters, interpreted as the adjusted mean attributable cost associated with an SUD diagnosis (principal or secondary) on the discharge record. The estimated total hospital cost per substance type was the mathematical combination (product) of the statistically estimated encounter count point estimate and encounter cost point estimate when the 95% CI for the cost point estimate was statistically greater than 0. Models controlled for patient demographic, clinical, and insurance characteristics as reported on the hospital discharge record: sex (male or female), age (in years), race/ethnicity (inpatient only; White, Black, Hispanic, Asian/Pacific Islander, Native American, other, missing), Clinical Classifications Software Revised classification of primary diagnosis (secondary diagnosis models only), indicators for each non-SUD comorbidity (eg, congestive heart failure), indicators for each secondary SUD diagnosis by substance type (eg, alcohol), hospital location and teaching status (rural, urban nonteaching, urban teaching), length of stay (inpatient only; in days), and disposition (routine discharge, transfer to short-term hospital, transfer other [eg, skilled nursing facility], home health care, against medical advice, died, unknown), and primary payer for the visit (Medicare, Medicaid, private, self-pay, no charge, other). Non-SUD comorbidities on the hospital discharge record were classified by the HCUP Comorbidity Index: congestive heart failure, valvular disease, pulmonary circulation disorders, peripheral vascular disease, hypertension, paralysis, other neurological disorders, chronic pulmonary disease, diabetes without chronic complications, diabetes with chronic complications, hypothyroidism, kidney failure, liver disease, chronic peptic ulcer disease, acquired immune deficiency syndrome, lymphoma, metastatic cancer, solid tumor without metastasis, rheumatoid arthritis or collagen vascular diseases, coagulation deficiency, obesity, weight loss, fluid and electrolyte disorders, blood loss anemia, deficiency anemias, psychoses, and depression.

^e^ Total estimated cost per substance type (eg, stimulant) is the sum of the estimated SUD cost per encounter (ED or inpatient) by SUD diagnosis type (principal or secondary) multiplied by the estimated number of encounters (when the estimated attributable cost of the diagnosis was significantly greater than 0—indicated by the modeled 95% CI of the marginal cost estimate being greater than 0). For example, for stimulant-related disorders total cost was calculated as follows from data in this table: ($2058 × 176 365) + ($385 × 606 166) + ($9690 × 54 898) + ($504 × 631 995) = $1447 million. The total estimated cost of all substance types is the sum of the total estimated cost of the individual substance types.

Model 2 included all encounters (any principal diagnosis). Controlling for principal diagnosis, model 2 compared total encounter cost among encounters with and without secondary SUD diagnoses. This model’s estimated marginal effect of secondary SUD diagnoses (eg, using Stata’s margins, dydx [alcohol]) was the estimated adjusted mean attributable cost of a SUD secondary diagnosis (Table 2) when the 95% CI for the marginal effect cost estimate was greater than 0. The estimated total hospital cost per substance type was the mathematical combination (product) of the statistically estimated encounter count point estimate and encounter cost point estimate. Descriptive data are shown in the eTable in the Supplement.

Through these models, the cost of an ED visit followed by inpatient admission with a principal diagnosis of heroin poisoning and secondary diagnoses including alcohol and cocaine dependence would be captured as follows: The majority of the encounter cost would be captured in model 1—owing to the principal SUD diagnosis—and assigned to this study’s opioid principal diagnosis cost category, after controlling for factors including the patient’s age, insurance type, length of inpatient stay, non-SUD comorbidities, and SUD secondary diagnoses. Any portion of the total encounter cost statistically associated with the alcohol and cocaine dependence secondary diagnoses—after controlling for the encounter principal diagnosis and other demographic, clinical, and insurance characteristics—would be captured in model 2 and assigned to this study’s alcohol and cocaine secondary SUD diagnosis cost categories, respectively.

## Results

This study examined a total of 124 573 175 hospital ED encounters and 33 648 910 inpatient encounters from the 2017 Healthcare Cost and Utilization Project Nationwide Emergency Department Sample and National Inpatient Sample. Of all hospital ED patient encounters, approximately 4% (based on survey-weighted point estimates: 4 987 039 / [4 987 039 + 119 586 136]) had an SUD diagnosis (principal or secondary), and of all hospital inpatient encounters, approximately 10% (based on survey-weighted point estimates: 3 381 115 / [3 381 115  + 30 267 795]) had an SUD diagnosis (principal or secondary) (Table 2). Alcohol-related disorders were the most common followed by opioid-related disorders. For some substances, far more discharge records had a secondary SUD diagnosis compared with a principal SUD diagnosis (eg, 606 166 ED encounters identified stimulant-related disorders as a secondary diagnosis vs 176 365 as a principal diagnosis; some encounters included both). The adjusted mean medical cost attributable to a principal SUD diagnosis among ED encounters was $1985 ($1893 to $2077). A secondary SUD diagnosis of any analyzed substance type on the ED record, with the exception of inhalant-related disorders, was associated with a mean increased encounter cost of $740 ($632 to $848). The adjusted mean medical cost attributable to a principal SUD diagnosis among inpatient encounters was $9693 (95% CI, $9361 to $10 025).

For most substances, each additional substance identified in a secondary SUD diagnosis on the hospital discharge record was associated with an increase in hundreds of dollars in total encounter cost. Secondary diagnoses of cannabis-, sedative-, or stimulant-related disorders were each associated with a higher inpatient cost (adding $165 [95% CI, $36 to $294], $374 [95% CI, $46 to $703], and $504 [95% CI, $294 to $713], respectively, to the encounter cost). The total estimated medical cost in hospitals attributable to SUD in 2017 was $13.2 billion. The cost by substance type ranged from $4 million (inhalant-related disorders) to $7.6 billion (alcohol-related disorders).

## Discussion

This study’s primary contribution is the estimated total annual SUD-associated medical cost in hospitals overall and by substance type using nationally representative US hospital data. This study also provides novel prevalence and associated cost estimates of principal and secondary SUD diagnoses during hospital encounters, offering a more complete picture of how hospital costs are associated with SUD compared with previous analyses.^2,3,5,15^ Polysubstance use was addressed in this study’s modeling approach by estimating the discrete associated cost of secondary SUD diagnoses by substance type; results suggest that for most substances, each additional substance identified in a secondary SUD diagnosis on the hospital discharge record was associated with an increase in hundreds of dollars in total encounter cost.

This study’s adjusted mean medical cost of encounters with principal SUD diagnosis is reasonably consistent with previous estimates after accounting for the professional fees and ambulance costs that were included here.^2,13^ This study’s estimated $13.2 billion medical cost associated with SUD represents a small fraction of all US hospital care expenditures ($1.1 trillion in 2017, or one-third of total health care spending^16^). Regardless of relative size, cost estimates by health condition are essential for decision-making about investments in prevention and treatment; for example, the cost of SUD treatment could be at least partially offset by a reduction in future SUD-related hospital care.

### Limitations

This study has some limitations. These estimates reflect medical costs incurred only in hospitals. Patients likely underreport substance use; therefore, results likely underestimate hospital costs attributable to SUD. These results do not address the cost of SUD borne by the patient and society in terms of lost quality of life and productivity. Approximately one-half of adults aged 18 years or older who reported past-year SUD also reported co-occurring mental illness.^17^ Statistical methods here explicitly controlled for physical and mental health comorbidities reported on the encounter record, but the estimates may not have completely excluded non-SUD costs. This study relied on *ICD-10-CM* codes to capture SUD identified during the hospital encounter; however, administrative records can inaccurately capture SUD.^18^ This study’s cost estimates controlled for demographic, clinical, and insurance characteristics reported on the hospital discharge record, but not some important factors likely associated with encounter cost among individuals with SUD, including homelessness.

## Conclusions

This study estimated the annual associated medical cost of SUD in US hospitals to be $13.2 billion. Direct medical cost estimates can help identify cost-effective ways to prioritize prevention and treatment. The cost of effective prevention and treatment may be substantially offset by a reduction in the high direct medical cost of SUD hospital care. Hospitalizations are critical opportunities to engage patients who are at high risk for overdose to prevent future overdoses, as hospital addiction care with referral to treatment increases outpatient SUD treatment engagement.^19,20^ This study’s results suggest that SUD creates substantial costs for hospitals and payers, yet few hospital patients receive SUD treatment services.^4,21,22^ Aligning incentives such that prevention cost savings accrue to payers and practitioners that are otherwise responsible for medical costs associated with SUD in hospitals and other health care settings may encourage prevention investment.

## Notes

Supplement. **eTable.** Descriptive Data
