# Supplementary material for: Assessment of Annual Cost of Substance Use Disorder in US Hospitals
Source: JAMA Netw Open. 2021 Mar 5;4(3):e210242. doi: 10.1001/jamanetworkopen.2021.0242 (PMC7936257; doi:10.1001/jamanetworkopen.2021.0242)
Supplement: Supplement. — eTable. Descriptive Data [file jamanetwopen-e210242-s001.pdf]

Supplemental Online Content

Peterson C, Li M, Xu L, Mikosz CA, Luo F. Assessment of annual cost of substance use disorder in US hospitals. *JAMA Netw Open*. 2021;4(3):e210242.
doi:10.1001/jamanetworkopen.2021.0242

eTable. Descriptive Data

This supplemental material has been provided by the authors to give readers additional information about their work.

© 2021 Peterson C et al. *JAMA Network Open.*

eTable. Descriptive Data

Characteristic	Emergency department visits				Inpatient admissions			
	Principal SUD diagnosis		Secondary SUD diagnosis		Principal SUD diagnosis		Secondary SUD diagnosis	
	Encounters (%)	Rate per 100,000 population	Encounters (%)	Rate per 100,000 population	Encounters (%)	Rate per 100,000 population	Encounters (%)	Rate per 100,000 population
Total	100.0	668	100.0	1,004	100.0	203	100.0	968
Age								
0-17	3.1	92	2.5	110	.7	6	1.3	54
18-44	57.0	1,062	57.0	1,596	44.8	254	39.0	1,053
45-64	35.1	905	34.1	1,324	45.2	354	42.7	1,598
65-74	3.8	275	4.7	519	7.2	160	11.3	1,200
75+	1.0	100	1.7	262	2.1	66	5.7	844
Sex								
Male	67.5	915	62.3	1,271	65.9	272	60.7	1,194
Female	32.5	427	37.7	745	34.1	136	39.3	749
Race/ethnicity								
White, non-Hispanic	NA	NA	NA	NA	66.6	219	62.5	978
Black, non-Hispanic	NA	NA	NA	NA	14.1	218	19.2	1,417
AI/AN, non-Hispanic	NA	NA	NA	NA	1.2	299	1.1	1,288
Asian/PI, non-Hispanic	NA	NA	NA	NA	.9	29	1.1	167
Hispanic	NA	NA	NA	NA	10.7	121	10.4	555
Other	NA	NA	NA	NA	3.2	NA	2.6	NA
Unknown	NA	NA	NA	NA	3.3	NA	3.2	NA
Non-SUD comorbidities, n								
0	61.7	412	49.7	499	15.1	31	16.3	158
1	24.1	161	26.9	270	22.2	45	19.6	189
2	9.3	62	13.0	130	21.5	44	19.2	186
3	3.4	22	6.1	61	17.1	35	16.3	158
4+	1.6	11	4.4	44	24.2	49	28.6	277
Length of stay, n days								
0	NA	NA	NA	NA	3.0	6	2.1	21
1	NA	NA	NA	NA	14.0	29	11.9	115
2	NA	NA	NA	NA	19.9	40	18.3	177
3	NA	NA	NA	NA	19.0	39	16.7	162
4+	NA	NA	NA	NA	44.2	90	51.0	494
Disposition								
Routine	88.3	590	85.4	858	71.4	145	67.8	656
Transfer to short-term hospital	1.4	10	3.1	31	1.9	4	2.1	21
Other transfers	5.1	34	6.5	65	11.6	24	13.8	134
Home health care	.2	1	.4	4	4.2	9	8.8	85

© 2021 Peterson C et al. *JAMA Network Open.*

Against medical advice	4.4	30	4.0	40	9.5	19	5.7	55
Died	.0	0	.1	1	1.4	3	1.8	17
Not admitted, destination unknown	.5	3	.5	5	0	0	0	0
Discharged alive, destination unknown	.0	0	NR	NR	NR	NR	.0	0
Primary payer								
Public (Medicare/Medicaid)	49.7	332	51.4	516	61.8	125	66.4	643
Private	20.0	134	20.0	201	22.3	45	20.1	195
Self-pay	25.6	171	24.0	241	11.3	23	9.2	89
No charge/Other	4.7	31	4.6	47	4.6	9	4.2	41
Location/teaching status of hospital								
Rural	11.1	74	15.4	155	8.5	17	8.0	77
Urban non-teaching	21.6	144	22.5	226	22.8	46	22.4	217
Urban teaching	67.3	449	62.1	623	68.6	139	69.7	674
Comorbidity conditions								
Congestive heart failure	.7	5	2.2	22	4.5	9	7.1	69
Valvular disease	.2	1	.6	6	1.4	3	2.3	23
Pulmonary circulation disease	.0	0	.1	1	.2	1	.7	7
Peripheral vascular disease	.2	1	.7	7	1.3	3	3.7	36
Hypertension	16.0	107	23.0	231	39.1	80	42.1	407
Paralysis	.1	1	.4	4	.9	2	2.3	22
Other neurological disorders	6.6	44	5.8	58	15.3	31	10.2	99
Chronic pulmonary disease	6.0	40	10.1	102	16.6	34	21.8	211
Diabetes w/o chronic complications	3.7	25	5.2	52	6.1	12	7.1	69
Diabetes w/ chronic complications	.9	6	2.2	22	5.3	11	9.5	92
Hypothyroidism	1.0	7	1.9	19	5.0	10	6.8	66
Renal failure	.5	3	1.6	17	4.3	9	8.2	79
Liver disease	1.7	11	3.9	39	10.7	22	13.5	131
Peptic ulcer disease with bleeding	.1	0	.2	2	1.0	2	1.1	10
Acquired immune deficiency syndrome	.3	2	.5	5	.5	1	.7	7
Lymphoma	.0	0	.1	1	.1	0	.4	4
Metastatic cancer	.0	0	.2	2	.4	1	1.5	15
Solid tumor w/out metastasis	.1	1	.3	4	1.0	2	1.5	14
Rheumatoid arthritis/collagen vas. dis.	.3	2	.7	7	1.2	2	2.0	20
Coagulopthy	.5	4	.9	9	14.6	30	10.0	97
Obesity	.7	5	2.2	22	6.9	14	11.3	109
Weight loss	.1	1	.4	5	5.8	12	7.3	71
Fluid and electrolyte disorders	4.6	31	7.2	72	36.5	74	34.3	332
Chronic blood loss anemia	.0	0	.1	1	1.1	2	1.6	16
Deficiency anemias	1.0	6	2.4	24	14.0	28	16.9	164
Psychoses	5.7	38	8.3	83	13.8	28	9.2	89
Depression	8.7	58	10.3	104	27.5	56	18.5	179

© 2021 Peterson C et al. *JAMA Network Open.*

Data source: Healthcare Cost and Utilization Project Nationwide Emergency Department Sample and National Inpatient Sample, 2017.
Abbreviations: AI/AN = American Indian / Alaska Native, NA = not applicable, NR = not reported (the relative standard error was over 30 percent or the standard error = 0; the value of the estimate was considered unreliable), PI = Pacific Islander, SUD = substance use disorder.
Notes: Encounters with a principal SUD diagnosis are not mutually exclusive from those with a secondary SUD diagnosis. SUD diagnosis rates by age, sex, and race are based on population size for those categories. SUD rates by comorbidities, length of stay, primary payer, and status of hospital are based on total population. Population data are from the 6/28/2019 update from the National Center for Health Statistics.

© 2021 Peterson C et al. *JAMA Network Open.*
